# Isolation and identification of *Clavibacter* made easy

**DOI:** 10.1128/aem.00871-26

**Published:** 2026-09-22

**Authors:** Nastaran Zamani, Zahra Sadeghi Mosavar, Ardavan Soleimani, Ebrahim Osdaghi

**Affiliations:** 1 Department of Plant Protection, College of Agriculture, University of Tehran 48424, Karaj, Iran; The University of Arizona, Tucson, Arizona, USA

**Keywords:** *Clavibacter michiganensis*, corynebacteria, coryneform bacteria, *Microbacteriaceae*, semi-selective medium

## Abstract

*Clavibacter* species are the most important gram-positive bacterial plant pathogens in terms of both disease severity and economic importance. As a group of regulated and quarantined plant pathogens, *Clavibacter* members are slow-growing organisms on general culture media, leading to difficulties in their isolation and identification. In this study, we developed an efficient, straightforward method for the isolation and identification of *Clavibacter* members from plant materials. First, a semi-selective medium was developed from a modification of conventional YPGA and YDC media, namely ClaYPGA and ClaYDC, respectively. This semi-selective medium contained four antibiotics, that is*,* colistin (100.0 mg/L), nalidixic acid (5.0 mg/L), sulfamethoxazole (5.0 mg/L), and trimethoprim (5.0 mg/L) for the inhibition of non-target bacterial growth, as well as natamycin (500.0 mg/L) for the inhibition of fungal growth. The ClaYPGA/ClaYDC medium supported the growth of all *Clavibacter* species while inhibiting the growth of almost all gram-negative bacteria, gram-positive *Bacillus* and *Streptomyces*, and fungi. Furthermore, ClaYPGA and ClaYDC did not affect the biological features, that is*,* cell density, and colony morphology of *Clavibacter* species compared to the basal YPGA and YDC. To increase the specificity of semi-selective medium, the primer pair ClaFor/ClaRev was designed based on the nucleotide sequence of a 259 bp DNA fragment found only in *Clavibacter* members. These PCR primers were strictly specific to *Clavibacter* species. The combination of semi-selective medium and ClaFor/ClaRev PCR primers increased the precision of the isolation and identification of *Clavibacter* species to such an extent that it has never been reported in the literature.

**IMPORTANCE:**

Regulated plant pathogenic bacteria that are subjected to quarantine restrictions play a vital role in the global trade of agricultural products. Plant protection authorities and quarantine inspectors need sensitive, dependable, cost-effective, and easy-to-use, straightforward methods to monitor the status of regulated bacterial agents either in the field or in transportation ports. *Clavibacter* species are the most destructive gram-positive bacterial plant pathogens. However, isolation and identification of these pathogens are affected by their slow-growing habit on general bacteriological culture media. Until 2015, only five *Clavibacter* lineages had been identified infecting alfalfa, maize, potato, tomato, and wheat. Within the past decade, however, the range of plant-associated *Clavibacter* members has been expanded to 13 standalone species. Selective or semi-selective culture media that have so far been developed for the isolation of *Clavibacter* targeted only the five original pathogens. Thus, application of those culture media nowadays would leave the expanded range of *Clavibacter* species untargeted. In this study, we aimed to develop a semi-selective medium to support the growth of all *Clavibacter* lineages while combating the growth of gram-negative bacteria, non-target gram-positive bacteria, and fungi. Furthermore, we designed a PCR primer strictly specific for *Clavibacter* members with no amplification in the other bacteria or any other living organism. Supplementing the results of semi-selective medium and specific PCR primers led to setting up a reliable, easy-to-use laboratory method for the isolation and identification of *Clavibacter* species.

## INTRODUCTION

Members of the corynebacterial genus *Clavibacter* (family *Microbacteriaceae*) are the most important gram-positive bacterial plant pathogens in terms of both disease severity and economic importance (1). *Clavibacter* was described in 1984, including several pathogenic members on alfalfa, maize, pepper, potato, tomato, and wheat (2). Before 1984, *Clavibacter* pathogens had been classified as members of *Corynebacterium* (3). All *Clavibacter* lineages have originally been classified as subspecies of *Clavibacter michiganensis sensu lato* in 1984; however, since 2018, the latter subspecies have been elevated to the species level based on DNA similarity indices (4). Currently, *Clavibacter* includes 13 standalone species, most of which are economically important plant pathogens and restricted agents around the globe (5–7).

For instance, *Clavibacter michiganensis,* the causal agent of tomato bacterial canker disease, is a globally widespread pathogen in the tomato industry. The bacterium is in the A2 quarantine list of the European and Mediterranean Plant Protection Organization (EPPO code: CORBMI; https://gd.eppo.int/taxon/CORBMI) and hence is subjected to strict regulations in all continents (8, 9). On the other hand, the potato pathogen *Clavibacter sepedonicus* is one of the most destructive bacterial pathogens in this crop (10). The Goss’s wilt pathogen of maize, *Clavibacter nebraskensis*, is also considered an emerging threat for maize production in North America, Mexico, and South Africa (11). Furthermore, five *Clavibacter* species infect small grain cereals in the *Poaceae* family, all considered emerging pathogens in American and Asian countries (7, 12). While the economic impact of the *Clavibacter* species infecting the *Poaceae* family remains to be elucidated, the pathogens of solanaceous crops, that is*, C. michiganensis* and *C. sepedonicus,* are considered a significant constraint on global seed trade (6, 10).

*Clavibacter* species are slow-growing organisms on general bacteriological culture media, leading to difficulties in their isolation and identification (13). Basal culture media, such as nutrient agar (NA) and Luria-Bertani agar (LB), are not suitable for the isolation of these bacteria from plant materials, since *Clavibacter* colonies would be outcompeted by saprophytic and epiphytic bacteria and fungi in the first 48 h post-incubation. Indeed, *Clavibacter* colonies usually need 4*–*6 days to be visible, which is long enough for saprophytes to cover the entire surface of the culture plates (1). It is highly recommended to use enriched culture media for the isolation and purification of plant pathogenic coryneform bacteria, that is*,* members of *Clavibacter*, *Curtobacterium*, and *Rathayibacter* (14). Commonly used nutritionally enriched culture media for the isolation of corynebacteria include YPGA (yeast extract 5.0 g, peptone 5.0 g, glucose 10.0 g, and agar 15.0 g in 1 L distilled water), NBY (nutrient broth 8.0 g, yeast extract 2.0 g, K_2_HPO_4_ 2.0 g, KH_2_PO_4_ 0.5 g, MgSO_4_ 0.25 g, glucose 5.0 g, and agar 15.0 g in 1 L distilled water), and YDC (yeast extract 10.0 g, dextrose/glucose 20.0 g, calcium carbonate 20.0 g, and agar 15.0 g in 1 L distilled water) (15). While the latter culture media promote rapid growth and multiplication of *Clavibacter* pathogens, their applicability in pathogen surveillance and diagnostic assays is still unsatisfactory. Indeed, enriched media simultaneously increase the growth rate of both target bacteria (*Clavibacter* species) and non-target organisms, such as saprophytic bacteria and fungi, which would lead to the overgrowth of the latter group (14). Economically important members of the genus, such as *C. michiganensis* and *C. sepedonicus,* are seed- and tuber-borne pathogens, respectively. In the latter cases, the primary diagnostic challenge lies in detecting them in seed/tuber lots to prevent their introduction into new regions, rather than in symptomatic field samples where bacterial loads are high, and isolation is relatively straightforward. On the other hand, the increasing number of new *Clavibacter* taxa within the last decade (7), which expanded the biological boundaries of the genus, highlights the importance of the availability of a genus-specific method to ease the isolation and identification of previously unknown *Clavibacter* infections.

To address these issues, selective or semi-selective media have been developed for the isolation of *Clavibacter* pathogens from plant materials, seed lots, and soil samples. In most cases, semi-selective media have been developed based on the original medium NBY (16), supporting the growth of only one *Clavibacter* lineage. Indeed, before the genomics era, it was supposed that only one *Clavibacter* lineage was associated with each host plant, that is*,* solanaceous vegetables and grasses. This is in contrast with the current expanding range of *Clavibacter* pathogens as revealed in the past two decades. For instance, it has long been thought that all *Clavibacter* strains associated with tomato, pepper, and eggplant belonged to *C. michiganensis sensu stricto*. However, current in-depth investigations revealed that at least four other *Clavibacter* species, such as *Clavibacter californiensis*, *Clavibacter capsici*, *Clavibacter lycopersici*, and *Clavibacter phaseoli,* could be associated with these crops (17). Furthermore, *Clavibacter tessellarius* was considered the sole causal agent of bacterial mosaic on small grain cereals for almost four decades. Only recently has it been clarified that, in addition to *C. tessellarius*, *Clavibacter davisi*, *Clavibacter rahimiani*, *Clavibacter vidaverae*, and *Clavibacter zhangzhiyongii* are also pathogenic on small grain cereals (7, 18). Hence, one could easily wonder if a semi-selective medium developed for one of these species could support the growth of the other accompanying lineages. While semi-selective media eased the isolation of originally described *Clavibacter* species, they could not be applied for isolation of the entire range of these pathogens. For instance, the BCT medium developed by Ftayeh et al. (19) targets only *C. michiganensis,* ignoring other species associated with tomato and pepper.

Thus, application of semi-selective media for the isolation of *Clavibacter* should be accompanied by advanced molecular techniques for the precise identification of these bacteria. Despite the economic importance of *Clavibacter* infections in agriculture and the ever-expanding genetic diversity of these pathogens within the past few decades, no satisfactory diagnostic molecular method covering all *Clavibacter* lineages has been available. Within the past three decades, a few PCR-based detection methods have been designed for the identification of *Clavibacter* species. However, the usefulness of these methods is negatively affected by the following two issues: first, these PCR techniques were developed when only five plant-associated *Clavibacter* pathogens were identified; thus, they are no longer considered an inclusive tool for the identification of all lineages of the genus. Second, none of these molecular techniques could strictly differentiate *Clavibacter* strains from taxonomically closely related non-target genera, such as *Curtobacterium*, *Microbacterium*, and *Rathayibacter* (13). Indeed, the only generic PCR primer for *Clavibacter* members is CMR16F1/CMR16R1, which was introduced before the beginning of the genomics era (20). The latter primer pair, which was designed based on the 16S rDNA sequence, directs the amplification of a 1,425 bp DNA fragment not only in *Clavibacter* species but also in several non-target organisms within the *Microbacteriaceae* family (6). All these gaps in the field of plant-associated *Clavibacter* highlight the need for a precise, comprehensive, and reliable method for the isolation and identification of these economically important organisms in agriculture.

The objectives of the present study were to (i) develop a semi-selective medium to support the growth of all *Clavibacter* lineages while combating the growth of gram-negative and non-target gram-positive bacteria, (ii) design a PCR primer strictly specific for *Clavibacter* members with no amplification in the other bacteria, and (iii) combine the results of semi-selective medium and specific PCR primers to set a reliable, easy-to-use laboratory method for the isolation and identification of *Clavibacter* species. Evaluation of the combined semi-selective medium and PCR assay developed in this study on a wide range of plant-associated bacteria showed that they could help to cost-effectively isolate and reliably identify *Clavibacter* species in a reasonable period of time.

## RESULTS

### Development of semi-selective medium

Preliminary screenings using nine antibiotics showed that ciprofloxacin and neomycin inhibited the growth of both gram-positive and gram-negative bacteria; thus, they were excluded from the subsequent experiments. Amikacin, imipenem, and meropenem did not provide satisfactory results in the screening tests. Based on the growth patterns of all tested bacteria, amikacin, imipenem, and meropenem were also considered inappropriate for the development of the selective medium for *Clavibacter* members. The bioassays led to the selection of colistin, nalidixic acid, sulfamethoxazole, and trimethoprim based on their ability to suppress the growth of gram-negative bacteria and non-target gram-positive bacteria, such as *Bacillus*, with no inhibitory effect on corynebacteria belonging to the *Microbacteriaceae* family. The results of the screening assays using the latter four antibiotics are detailed in Table S1. The optimal concentrations of these antibiotics were determined as 100.0 mg/L for colistin, 5.0 mg/L for nalidixic acid, 5.0 mg/L for sulfamethoxazole, and 5.0 mg/L for trimethoprim. On the other hand, natamycin showed effective inhibition of fungal growth without adverse effects on bacterial growth. Therefore, it was selected as the fungicide agent at a concentration of 500.0 mg/L. To finalize the new medium, YPGA and YDC were considered the basal media. Antimicrobial agents were added to the basic ingredients of these media after autoclaving. The new media that resulted from the modifications of YPGA and YDC were named as ClaYPGA and ClaYDC, respectively. Detailed ingredients of the ClaYPGA and ClaYDC media are listed in Table S2. Selectivity of ClaYPGA and ClaYDC media was evaluated using a broader collection of bacterial strains as detailed in Table 1 and Table S3.

**TABLE 1:** *Clavibacter* strains used in this study, their origin, and results of their growth on ClaYPGA/ClaYDC medium and PCR using ClaFor/ClaRev primers

Taxon	Strain	Host of isolation	Pathogenic on	Colony color	Growth on ClaYPGA and ClaYDC	*Clavibacter*-specific PCR
*Clavibacter michiganensis*	ICMP 2550^T^	Tomato	Tomato	Yellow	Positive	Positive
*Clavibacter michiganensis*	CFBP 9078	Tomato	Tomato	Pink	Positive	Positive
*Clavibacter insidiosus*	CFBP 2404^T^	Alfalfa	Alfalfa	Yellow	Positive	Positive
*Clavibacter nebraskensis*	CFBP 2405^T^	Maize	Maize	Orange	Positive	Positive
*Clavibacter lycopersici*	CFBP 8615^T^	Tomato	Non-pathogenic	Peach color	Positive	Positive
*Clavibacter tessellarius*	CFBP 9248	Wheat	Small grain cereals	Orange	Positive	Positive
*Clavibacter tessellarius*	CFBP 9075	Wheat	Small grain cereals	Orange	Positive	Positive
*Clavibacter tessellarius*	CFBP 9076	Wheat	Small grain cereals	Orange	Positive	Positive
*Clavibacter tessellarius*	CFBP 9233	Wheat	Small grain cereals	Orange	Positive	Positive
*Clavibacter tessellarius*	CFBP 9398	Wheat	Small grain cereals	Orange	Positive	Positive
*Clavibacter tessellarius*	Sh3075	Wheat	Small grain cereals	Orange	Positive	Positive
*Clavibacter tessellarius*	CFBP 3496^T^	Wheat	Small grain cereals	Orange	Positive	Positive
*Clavibacter zhangzhiyongii*	CFBP 9073	Barley	Small grain cereals	Orange	Positive	Positive
*Clavibacter zhangzhiyongii*	CFBP 9074	Oat	Small grain cereals	Orange	Positive	Positive
*Clavibacter zhangzhiyongii*	CFBP 9135	Barley	Small grain cereals	Orange	Positive	Positive
*Clavibacter zhangzhiyongii*	CFBP 9348	Wheat	Small grain cereals	Orange	Positive	Positive
*Clavibacter rahimiani*	CFBP 9069^T^	Wheat	Small grain cereals	Peach color	Positive	Positive
*Clavibacter rahimiani*	CFBP 9347	Wheat	Small grain cereals	Peach color	Positive	Positive
*Clavibacter vidaverae*	CFBP 9070^T^	Wheat	Small grain cereals	Pale orange	Positive	Positive
*Clavibacter vidaverae*	CFBP 9243	Wheat	Small grain cereals	Pale orange	Positive	Positive
*Clavibacter davisi*	CFBP 9071^T^	Oat	Small grain cereals	Deep yellow	Positive	Positive
*Clavibacter davisi*	CFBP 9077	Oat	Small grain cereals	Deep yellow	Positive	Positive

### Efficiency of the semi-selective medium

Both ClaYPGA and ClaYDC media inhibited the growth of almost all non-target bacterial species, as shown in Fig. S1 and Table S3. Besides the inhibition of almost all gram-negative bacteria, no growth was recorded for the omnipresent soil-dwelling bacterial species *Bacillus subtilis*, *Bacillus velezensis*, *Bacillus cereus*, *Paenibacillus polymyxa*, *Paenibacillus* sp., and *Streptomyces*. The growth of all enterobacterial species was also totally inhibited on the ClaYPGA and ClaYDC media. More specifically, the fast-growing *Pantoea* and *Enterobacter* species, which are the most frequent gram-negative agents interfering with the isolation of slow-growing plant pathogenic corynebacteria, were inhibited, as illustrated in Fig. S1. The new ClaYPGA and ClaYDC media also inhibited the growth of all *Xanthomonas* species. A few exceptions were recorded among the gram-negative bacteria. *Burkholderia phenazinium* strain CFBP 4793^T^, *Burkholderia gladioli* strain CFBP 9085, *Burkholderia pyrrocinia* strain CFBP 4794^T^, *Burkholderia cenocepacia* strain CFBP 8617, *Burkholderia cepacia* strain CFBP 2227^T^, *Paraburkholderia graminis* strain CFBP 6734^T^, and *Pseudomonas tolaasii* strains CFBP 8707 were the only gram-negative bacterial strains that showed a trace of colony growth on ClaYPGA. However, the growth speed and colony size of the latter species on ClaYPGA and ClaYDC were significantly lower than those observed on the basal unmodified YPGA and YDC media.

**Fig 1:**
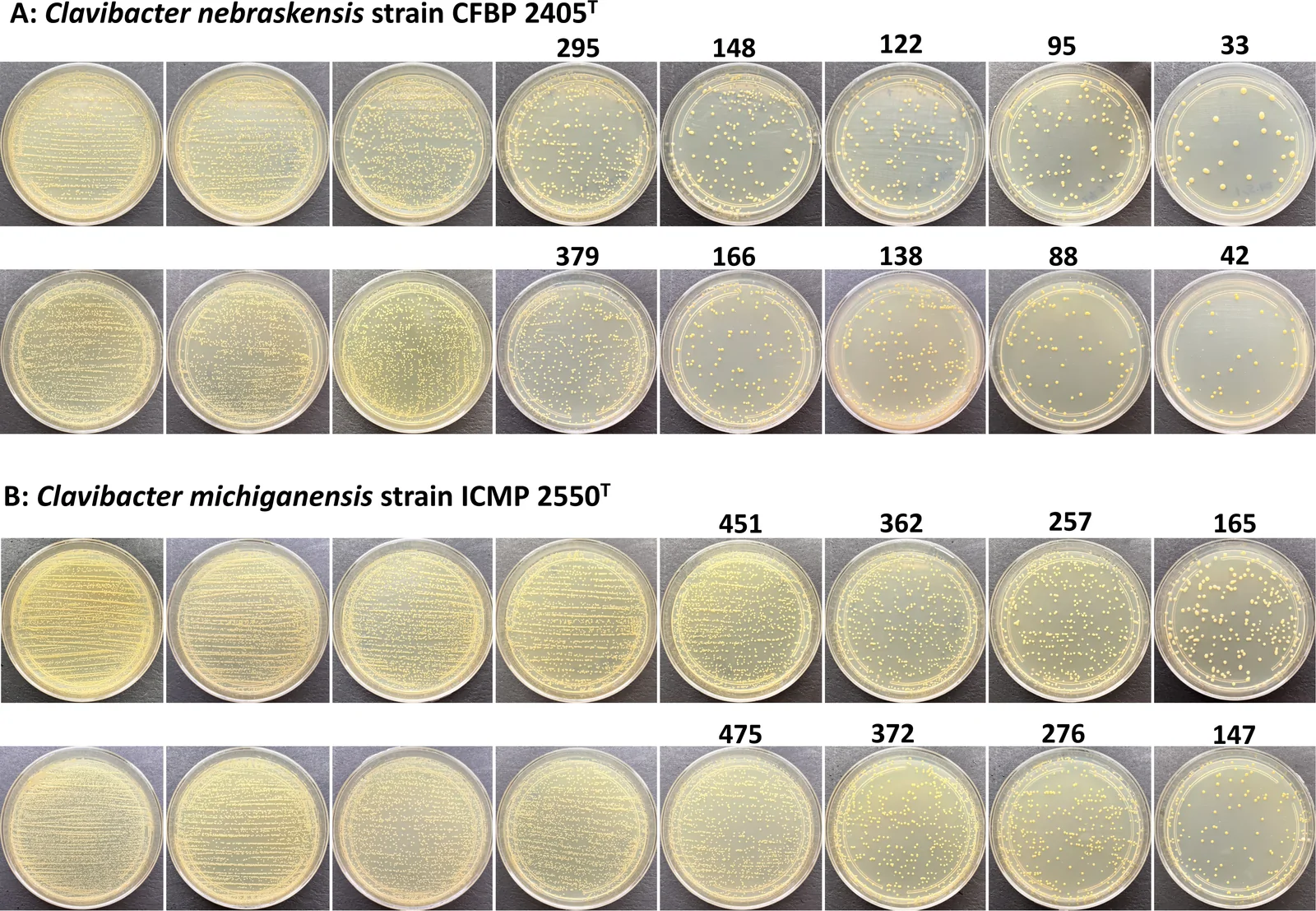
Growth pattern and morphological features, that is*,* colony size and color, of *C. nebraskensis* CFBP 2405^T^ (**A**) and *C. michiganensis* ICMP 2550^T^ (**B**) on YPGA (top row) and ClaYPGA (bottom row) media. Bacterial cells of the two strains were serially diluted in sterile distilled water at a 1:1 ratio; 20 µL of the resulting suspension was spread onto YPGA and ClaYPGA plates and incubated at 25°C–27°C for 7 days. The number and morphology of colonies*,* that is, size, shape, and color, did not differ between the basal YPGA and modified semi-selective ClaYPGA medium. The numbers on each plate represent average colony-forming units calculated from all repeats of the experiments.

As for the growth patterns of corynebacterial plant pathogens on ClaYPGA and ClaYDC media, all members of the nine *Clavibacter* species evaluated in this study showed normal growth, as shown in Fig. S2. In order to assess if the ClaYPGA and ClaYDC media have any negative impact on the growth speed, colony size, and vigor of the *Clavibacter* strains, serial dilutions from a water suspension of type strains of *C. michiganensis* ICMP 2550^T^ and *C. nebraskensis* CFBP 2405^T^ were spread on these media, and the growth parameters were monitored up to 10 days post-incubation. As illustrated in Fig. 1A and B, the number and size of colonies were highly similar on ClaYPGA and YPGA media in both bacterial species. Thus, the antibacterial agents integrated in ClaYPGA medium did not affect the growth pattern, morphology, and vigor of *Clavibacter* members.

Besides *Clavibacter* species that are easily raised on ClaYPGA and ClaYDC media, other corynebacterial genera, such as *Curtobacterium* and *Rathayibacter*, showed variable growth patterns on these media, as shown in Fig. S2. We assessed the growth of at least four *Rathayibacter* species and three *Curtobacterium* species on ClaYPGA and ClaYDC media. Type strains of *Rathayibacter iranicus* CFBP 807^T^, *Rathayibacter rathayi* CFBP 2406^T^, and *Rathayibacter tritici* CFBP 1385^T^, as well as the *Rathayibacter* strains CFBP 9124, CFBP 9072, and CFBP 9136, which are hypothetically novel species, showed normal growth on both ClaYPGA and ClaYDC media, comparable to the growth pattern of *Clavibacter* species. However, variable results were recorded in the growth of *Curtobacterium* species. For instance, growth of the common bean pathogen *Curtobacterium flaccumfaciens* pv. *flaccumfaciens* strain ICMP 22053 and *Curtobacterium aurantiacum* strain ICMP 22071, as well as the tulip pathogen *Curtobacterium flaccumfaciens* pv. *oortii* strain ICMP 2632^PT^, was severely inhibited on ClaYPGA. However, other *Curtobacterium* strains had more or less normal growth on both ClaYPGA and ClaYDC media as detailed in Table S3. Taken together, ClaYPGA and ClaYDC media developed in this study were shown to be strictly selective against gram-negative bacteria and non-corynebacterial gram-positive species; however, they had semi-selective behavior against non-*Clavibacter* corynebacterial species. Thus, we refer to ClaYPGA and ClaYDC as semi-selective media for *Clavibacter* members.

### *Clavibacter*-specific PCR primers

**Fig 2:**
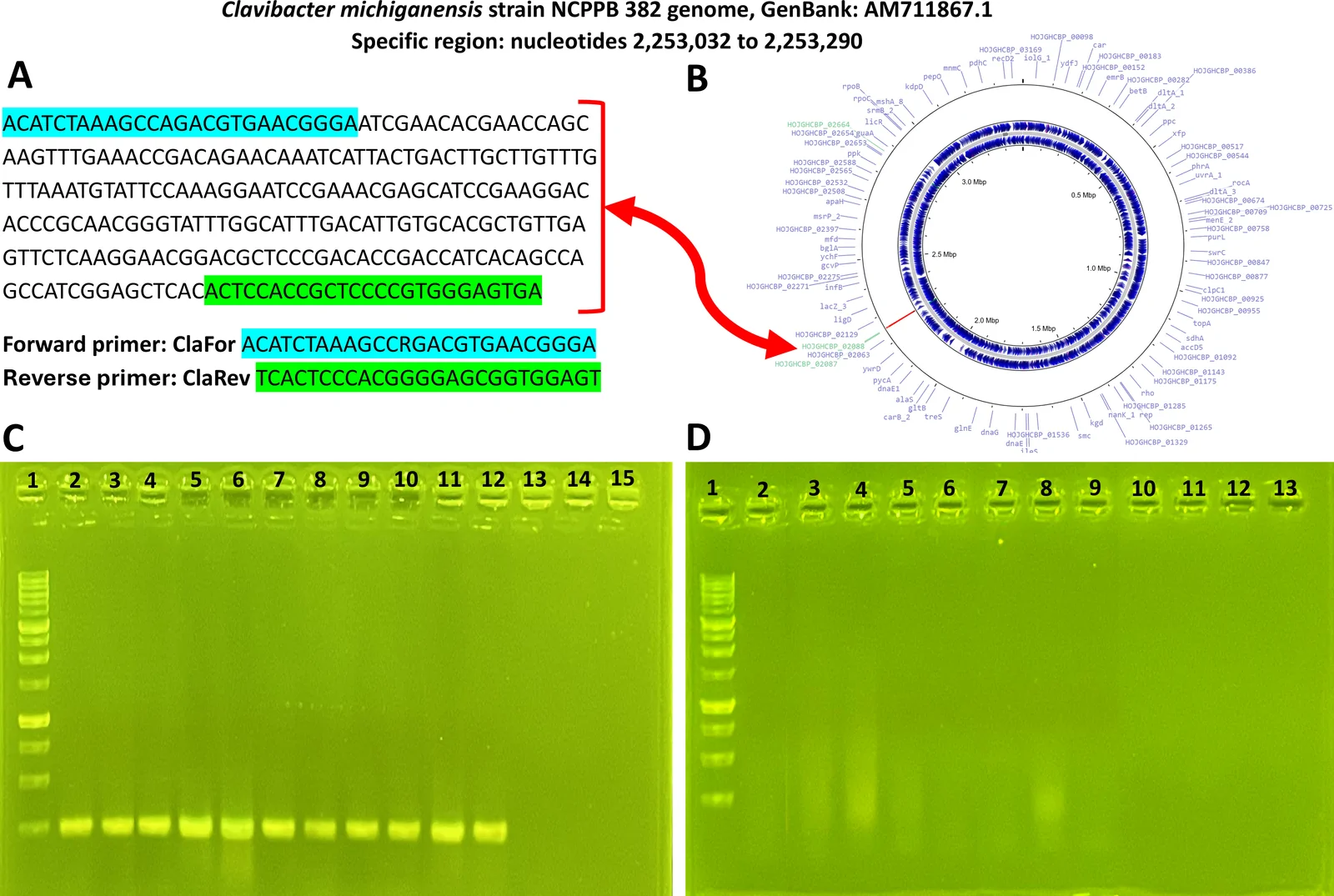
Nucleotide sequence of the 259-bp *Clavibacter*-specific DNA fragment used for designing ClaFor/ClaRev PCR primers (**A**), and genomic position of the DNA fragment on the complete genome sequence of *C. michiganensis* strain NCPPB 382 (GenBank: AM711867.1: nucleotides 2253032*–*2253290) (**B**). Agarose gel electrophoresis of PCR products using ClaFor/ClaRev primers on representative strains of gram-positive (**C**) and gram-negative (**D**) bacterial species. The first well in each gel is the DNA ladder. Wells 2*–*15 in panel C: (2 and 3) *C. michiganensis* ICMP 2550^T^ and CFBP 9078, (4) *C. insidiosus* CFBP 2404^T^, (5) *C. nebraskensis* CFBP 2405^T^, (6) *C. lycopersici* CFBP 8615^T^, (7) *C. tessellarius* CFBP 3496^T^, (8) *C. rahimiani* CFBP 9069^T^, (9) *C. vidaverae* CFBP 9070^T^, (10) *C. davisi* CFBP 9071^T^, (11 and 12) *C. zhangzhiyongii* CFBP 9073 and CFBP 9074, (13) *Rathayibacter rathayi* CFBP 2406, (14) *Curtobacterium flaccumfaciens* pv. *flaccumfaciens* ICMP 22057, and (15) *Bacillus cereus* PTCC 1015. Wells 2*–*13 in panel D: (2) *Acidovorax citrulli* CFBP 4459^T^, (3) *Burkholderia cepacia* CFBP 2227^T^, (4) *Dickeya dadantii* CFBP 9238, (5) *Pantoea stewartia* ICMP 24703, (6) *Pantoea ananatis* CFBP 9125, (7) *Pantoea agglomerans* ICMP 24706, (8) *Pseudomonas agarici* ICMP 24736, (9) *Pseudomonas tolaasii* CFBP 2068^T^, (10) *Serratia marcescens* CFBP 9107, (11) *Xanthomonas oryzae* pv. *oryzicola* CFBP 2286^PT^, (12) *Xanthomonas euvesicatoria* pv. *euvesicatoria* ICMP 22095, and (13) *Xanthomonas translucens* pv. *undulosa* CFBP 8908. The expected 259 bp DNA fragment was amplified only in *Clavibacter* strains.

*In silico* analyses of 489 *Clavibacter* genome resources resulted in the identification of a 259 bp DNA fragment, which was present in all strains of the genus. The latter DNA fragment was absent not only in all non-*Clavibacter* coryneform bacteria within the *Microbacteriaceae* family but also in all other gram-positive and gram-negative bacteria. A comprehensive BLASTn search in the NCBI GenBank database showed that the 259 bp target DNA fragment was also absent in all other living organisms, such as fungi, plants, and animals, as well as viruses (Fig. S3). The PCR primers ClaFor (ACATCTAAAGCCRGACGTGAACGGGA) and ClaRev (TCACTCCCACGGGGAGCGGTGGAGT) were designed to cover the entire 259-bp length of the target DNA, as shown in Fig. 2A and B. *In silico* PCR using NCBI Primer BLAST service showed that the ClaFor/ClaRev primer pair could direct the amplification of a 259-bp DNA fragment only in *Clavibacter* strains. To confirm the specificity of the ClaFor/ClaRev primer pair in wet-lab conditions, a collection of plant-associated bacteria covering both gram-positive and gram-negative domains was subjected to PCR assays. The bacterial collection used in the latter assay was the same as used for the development of semi-selective medium (Table S3). Parallel to the results of *in silico* PCRs, the expected 259 bp DNA fragment was amplified only in *Clavibacter* strains. Nevertheless, no amplification was observed in taxonomically closely related bacteria, such as *Curtobacterium*, *Rathayibacter*, and *Mycetocola saprophilus*; gram-negative bacteria; or representative fungal species (Fig. 2C and D).

### *In situ* pertinence of combined semi-selective medium and specific PCR

Based on the results obtained from the semi-selective medium and specific PCRs, the combination of the two techniques would significantly ease isolation and identification of *Clavibacter* members. Thus, bioassays were designed to isolate *Clavibacter* from both naturally infected plant specimens and randomly selected non-host plants in two independent experiments.

**Fig 3:**
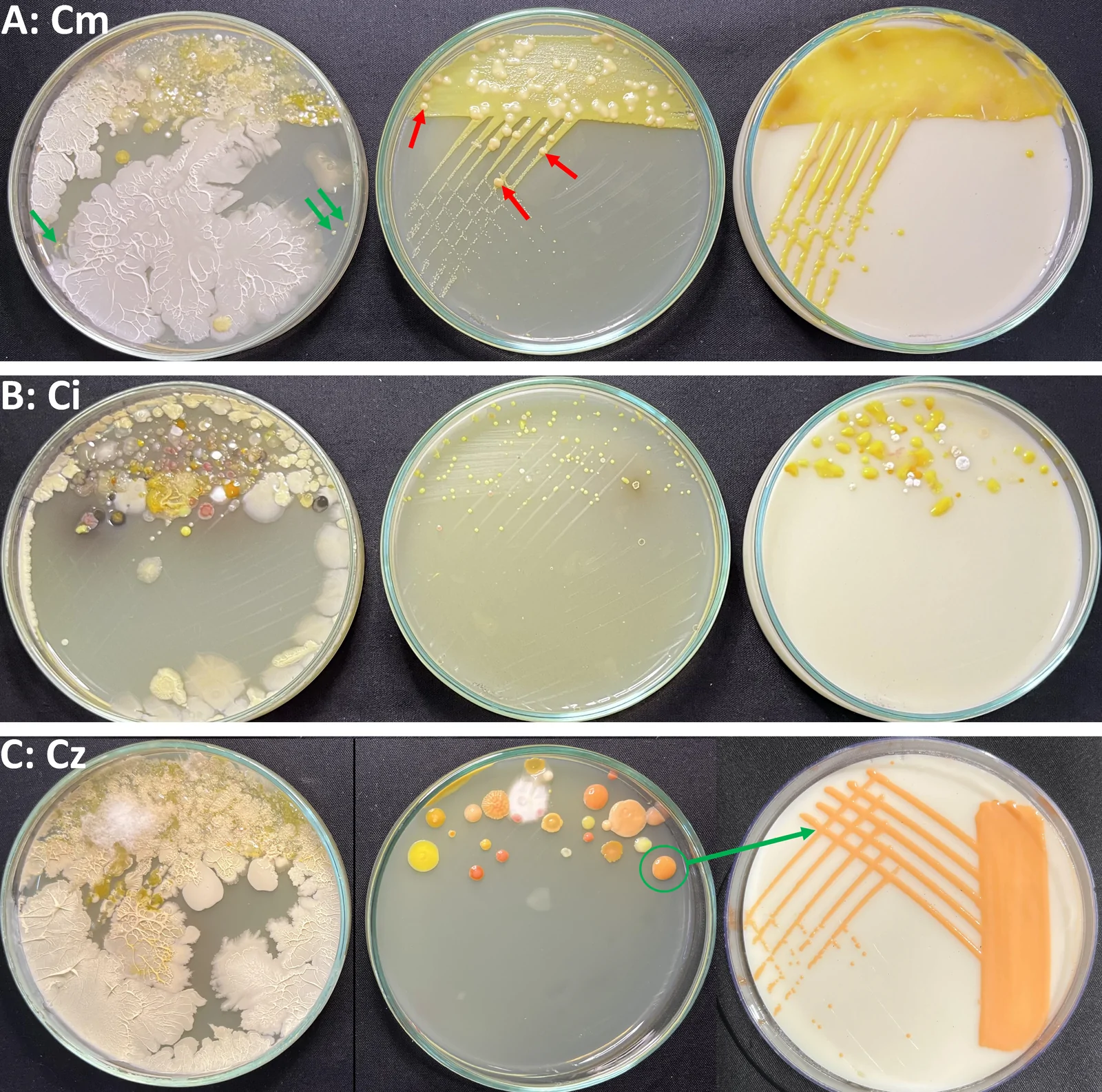
Isolation of *C. michiganensis* (**A**), *C. insidiosus* (**B**), and *C. zhangzhiyongii* (**C**) from naturally infected tomato, alfalfa, and cucumber plants, respectively. Smashed tissues of the specimens were suspended in sterile distilled water and streaked on YPGA (left column), ClaYPGA (middle column), and ClaYDC (right column). The plates were incubated at 25°C*–*27°C for 5*–*7 days. A *C. zhangzhiyongii* strain was isolated from cucumber leaves possessing orange colonies on YDC and ClaYDC media. Green arrows indicate target colonies (*Clavibacter*), while red arrows show non-*Clavibacter* colonies. Cm, *Clavibacter michiganensis*; Ci, *Clavibacter insidiosus*; Cz, *Clavibacter zhangzhiyongii*.

In the first experiment, plating the water suspension of tomato plants naturally infected with *C. michiganensis* on YPGA, YDC, ClaYPGA, and ClaYDC media resulted in the growth of yellow-pigmented colonies of the pathogen on all four media 4*–*6 days post-incubation. However, the colonies of *C. michiganensis* on YPGA were totally outcompeted by undesired bacterial and fungal saprophytes. On ClaYPGA and ClaYDC media, however, only colonies of *C. michiganensis* were observed along with a number of negligible gram-positive corynebacterial colonies, while the growth of all gram-negative bacteria, *Bacillus* and *Streptomyces* species, and fungi was inhibited (Fig. 3A). Similar results were obtained in the isolation of the slow-growing alfalfa pathogen *Clavibacter insidiosus* from naturally infected plants (Fig. 3B). Colonies of *C. insidiosus* on YPGA plates have rarely been noticeable among the other bacterial and fungal saprophytes. However, plating the same plant extract on ClaYPGA and ClaYDC media resulted in the growth of yellow-pigmented colonies resembling those of the alfalfa bacterial wilt pathogen. In both assays, including tomato and alfalfa specimens, dominant circular domed shiny yellow colonies were subjected to direct colony PCR using the ClaFor/ClaRev primer pair, which resulted in the amplification of the expected 259 bp DNA fragment. Occasional gram-positive colonies seen on ClaYPGA and ClaYDC media, for example*,* pink-colored colonies in the tomato assay (marked with red arrows in Fig. 3A), were also assessed using PCR, while no DNA amplification was observed in the latter tests.

In another set of bioassays, we assessed the pertinence of ClaYPGA and ClaYDC media and ClaFor/ClaRev primer pair for the isolation and identification of potential *Clavibacter* strains from non-hosts and irrelevant sources such as rhizosphere soils and aerial parts of asymptomatic monocotyledonous and dicotyledonous plants (i.e., annual crops, vegetables, and ornamentals) as detailed in Fig. S4. In all cases, microbial load on ClaYPGA and ClaYDC media was far less than that observed on YPGA and YDC media. Almost all bacterial colonies observed on ClaYPGA and ClaYDC plates were gram-positive, while a combination of gram-negative, gram-positive, and fungal growth was observed on YPGA and YDC plates. The gram-positive bacteria raised on ClaYPGA and ClaYDC media were morphologically resembling those of coryneform bacteria with circular domed shiny colonies, as shown in Fig. 3A through C. Representatives of all colony types grown on ClaYPGA and ClaYDC media were purified with re-streaking on the same media, and the resulting bacterial isolates were subjected to PCR using the ClaFor/ClaRev primer pair. The latter PCR primers directed the amplification of the expected 259-bp DNA fragment only in one of the orange-pigmented bacterial isolates, resulting from plating cucumber leaves on ClaYPGA and ClaYDC media, as shown in Fig. 3C. While streaking the water suspension of the cucumber leaf sample on YPGA and YDC plates resulted in the growth of saprophytic fungi and fast-growing bacteria, the ClaYPGA and ClaYDC plates contained a small number of colorful domed circular colonies possessing typical characteristics of corynebacteria. Further investigations were performed to clarify the precise taxonomic position of the *Clavibacter* strain isolated from cucumber leaves. Phylogenetic analyses using the sequences of the *gyrB* gene in the strain isolated in this study and representative members of *Microbacteriaceae* showed that the strain isolated from cucumber belonged to *Clavibacter zhangzhiyongii,* as shown in Fig. S5.

## DISCUSSION

*Clavibacter* species are the most important and destructive gram-positive bacterial plant pathogens all over the world. In this study, we developed an efficient, straightforward method for the isolation and identification of *Clavibacter* members from plant materials and soil samples. First, the semi-selective media ClaYPGA and ClaYDC were developed from modification of YPGA and YDC, respectively, by adding antibiotic agents into the basal ingredients of the latter media. These semi-selective media supported the growth of all *Clavibacter* species while inhibiting the growth of almost all gram-negative bacteria, gram-positive *Bacillus* and *Streptomyces* strains, and fungi. Modification of YPGA and YDC into ClaYPGA and ClaYDC did not have any adverse effect on the biological features, such as growth speed, cell density, and colony morphology of *Clavibacter* species, as proved by spreading type strains of *C. michiganensis* ICMP 2550^T^ and *C. nebraskensis* CFBP 2405^T^ onto these media. Subsequently, we designed a pair of PCR primers, which were strictly specific to *Clavibacter* species. The primer pair ClaFor/ClaRev directed the amplification of a 259-bp DNA fragment only in *Clavibacter* members, but neither in taxonomically closely related genera in the *Microbacteriaceae* family nor in any gram-negative bacteria, fungi, viruses, and plant species. The combination of ClaYPGA and ClaYDC semi-selective media and ClaFor/ClaRev PCR primers would increase the precision of the isolation and identification of *Clavibacter* species to an extent that has never been reported in the literature.

Semi-selective or selective media for isolation and detection of corynebacterial plant pathogens have long been used for both experimental and industrial purposes. For instance, Gross and Vidaver (16) developed a selective agar medium (CNS) for the isolation of *C. nebraskensis* from maize tissue and soil. Modified CNS agar developed by Vidaver and Davis (21) was a semi-selective medium on which *C. michiganensis* colonies appeared in 6*–*7 days. In contrast, neither *C. sepedonicus* nor *C. insidiosus* grew on the CNS-selective medium (22). On SCM agar developed by Fatmi and Schaad (23), gray-to-black speckled colonies of *C. michiganensis* are formed. Modification of SCM agar into m-SCM agar yielded clear colonies with yellow flecks, which appear 7*–*9 days post-incubation (24). Semi-selective media either alone or in combination with an immunofluorescence colony-staining procedure are also available for detection of the ring rot pathogen in naturally infected symptomless potato tubers (25, 26). However, development of these media was mainly focused on the major pathogens, such as *C. michiganensis*, *C. sepedonicus*, and *C. nebraskensis,* while the minor pathogens, such as *C. davisi*, *C. insidiosus*, *C. rahimiani*, *C. tessellarius*, *C. vidaverae*, and *C. zhangzhiyongii,* were almost entirely ignored in the investigations. Thus, isolation of the latter pathogens infecting alfalfa and small grain cereals, respectively, has always been a technical challenge for plant protection authorities and quarantine inspectors.

On the other hand, all the above-mentioned selective or semi-selective media narrowed down the range of selectivity to one lineage/species of *Clavibacter*. This strict specificity had advantages in field surveys and quarantine inspections, which could clearly address the problem as far as a specified pathogen is concerned. However, overconcentration of these media onto one bacterial species could result in false-negative outcomes, specifically when the evaluated plant material is infected with a novel or previously unreported lineage of *Clavibacter*. In this frame, Ftayeh et al. (19) noted that all previously published semi-selective media, that is, D2, KBT, D2ANX, SCM, m-SCM, CMM1, and mCNS, yielded false-negative results in the isolation of *C. michiganensis*. Thus, they have developed a new selective and highly sensitive medium for isolation of the tomato bacterial canker pathogen from seed and latently infected plants and named the new medium BCT. Recent expansions in the phenotypic range of tomato bacterial canker pathogens highlighted the need for reconsideration of the pertinence of all selective media, including BCT. Indeed, a new pink-pigmented tomato-pathogenic variant of *C. michiganensis* has recently been described (27). Applicability of BCT for the isolation of this new variant and phenotypic features of this variant, such as colony color and morphology, on the mentioned medium remain to be investigated.

*Clavibacter*-specific culture media have long been developed based on some presumptions that have later been challenged by new findings in the biodiversity of corynebacterial plant pathogens. Indeed, before the genomic era, all *Clavibacter* lineages were classified in five distinct subspecies of *C. michiganensis sensu lato* infecting alfalfa, maize, potato, tomato, and wheat (13). Until 2015, no information was available on non-pathogenic lineages of *Clavibacter* in the literature. In a few cases, when non-pathogenic strains of the genus were isolated from solanaceous vegetables, they have tentatively been classified as *C. michiganensis* subsp. *michiganensis* (17). Only within the past decade, genome sequence-based phylogenomics analyses have shed light on the biodiversity of *Clavibacter* lineages. For instance, it has been shown that besides *C. michiganensis*, four taxonomically distinct *Clavibacter* species—either pathogenic or non-pathogenic—were also associated with solanaceous vegetables. These new taxa include *C. californiensis*, *C. capsici*, *C. lycopersici*, and *C. phaseoli* (6). None of the latter lineages were known when semi-selective media were being developed for the tomato bacterial canker pathogen. Bacterial mosaic of small grain cereals also has the same issue. From the 1970s to the early 2020s, it was thought that bacterial mosaic disease was caused by *C. tessellarius*. However, recent investigations showed that four other *Clavibacter* species, including *C. davisi*, *C. rahimiani*, *C. vidaverae*, and *C. zhangzhiyongii*, also could induce bacterial mosaic on different small grain cereals such as wheat, barley, and oat (7). Considering the current genetic diversity of *Clavibacter* species, no genus-specific culture media are available to support the growth of all biological lineages within the genus. Taken together, the semi-selective media ClaYPGA and ClaYDC developed in this study are, on one hand, specific enough to suppress the growth of non-target gram-negative bacteria, as well as *Bacillus* and *Streptomyces* species. On the other hand, they are broad-spectrum to such an extent that they could be applied for isolation of all *Clavibacter* lineages regardless of their taxonomic position, pathogenicity, and habitat.

The *Clavibacter*-specific primer pair ClaFor/ClaRev developed in this study would complement the selectivity of ClaYPGA and ClaYDC media. While genus-specific PCR primers are available for the identification of economically important gram-negative plant pathogenic bacteria, such as *Xanthomonas* and *Pseudomonas* members (28, 29), such PCR primers have rarely been introduced for *Clavibacter*. The few available PCR primers for the identification of *Clavibacter* members are inclusive enough to cover all lineages of the genus (6). However, they are not exclusive for differentiating *Clavibacter* strains from taxonomically closely related genera such as *Curtobacterium*, *Microbacterium*, and *Rathayibacter* (30). Recently, a comprehensive *in silico* analysis of available PCR primers for *Clavibacter* showed that the primer pairs CMR16F1/CMR16R1, PSA-4/PSA-R, and Clav-F/Clav-R are generic for all *Clavibacter* species, as well as other genera within the *Microbacteriaceae* family (6). Thus, none of these primer pairs could be reliably used for the precise identification of *Clavibacter* members. This would be more challenging when one tries to isolate *Clavibacter* strains from unusual sources, such as non-host plants and environmental specimens. Isolation and identification of the orange-pigmented *Clavibacter* strain from cucumber leaves in this study supported the pertinence and usefulness of the co-application of ClaYPGA medium and ClaFor/ClaRev primers. Indeed, in the absence of such semi-selective medium and strictly specific PCR primers, it would be very time-consuming—if not impossible—to isolate and identify a *Clavibacter* strain from such an irrelevant plant species.

The *Clavibacter* strain isolated from cucumber leaves was identified as *C. zhangzhiyongii* based on the phylogeny of the *gyrB* housekeeping gene. *Clavibacter zhangzhiyongii* was isolated for the first time from barley seeds in China and shown to be pathogenic on this plant (31). The bacterium has also been isolated from barley and oat plants in Iran (32). While the host range of *C. zhangzhiyongii* within the *Poaceae* family has recently been investigated (18), its pathogenicity on a plant species beyond small grain cereals remains uninvestigated. We did not assess the pathogenicity of the *C. zhangzhiyongii* strain on cucumber plants, which could be the goal of another independent investigation.

In conclusion, the semi-selective medium developed in this study would ease the isolation of *Clavibacter* members from not only their known host plants but also irrelevant non-host plant species as well as environmental specimens. This semi-selective medium, either in ClaYPGA or ClaYDC formulation, supports the growth of only *Clavibacter* species and a few taxonomically closely related lineages. We intentionally kept the selectivity range of ClaYPGA and ClaYDC media slightly beyond *Clavibacter* boundaries to guarantee that no *Clavibacter* lineage, even those that have not yet been identified, would be missed in the isolation procedure. Furthermore, the *Clavibacter*-specific PCR primers ClaFor/ClaRev would complement the selectivity of ClaYPGA and ClaYDC media, where the selectively grown coryneform bacterial colonies on these media could easily be screened with a conventional PCR. It is noteworthy that the combination of ClaYPGA/ClaYDC media and ClaFor/ClaRev PCR primers would be more suitable for the isolation and identification of *Clavibacter* strains from atypical sources and non-host plant species where the taxonomic status of the target strains is previously unknown. For specific pathogens such as *C. michiganensis*, a set of sophisticated DNA sequence-based detection methods has already been reported in the literature, as reviewed recently (6). The results of the present study, on one hand, would increase the accuracy and reliability of preliminary screenings for the identification of plant-associated *Clavibacter* lineages. On the other hand, they would pave the way for investigations on the biodiversity and taxonomic range of the most important plant pathogenic gram-positive bacterial genus in the 21st century’s agriculture.

## MATERIALS AND METHODS

### Biological resources and basic culture media

Reference strains of *Clavibacter* species*,* such as *C. davisi*, *C. insidiosus*, *C. lycopersici*, *C. michiganensis*, *C. nebraskensis*, *C. rahimiani*, *C. tessellarius*, *C. vidaverae*, and *C. zhangzhiyongii*, were provided by CIRM-CFBP (International Center of Microbial Resource, French Collection for Plant-associated Bacteria, INRAE, https://cirm-cfbp.fr/page/Home), as listed in Table 1. Additionally, type/pathotype/reference strains of plant-associated bacteria covering both gram-positive and gram-negative groups were also included in the biological assays as controls (Table S3). These bacterial strains were either provided by CIRM-CFBP or ICMP (International Collection of Microorganisms from Plants, Auckland, New Zealand; https://scd.landcareresearch.co.nz/) culture collections. A few strains were also obtained from a local culture collection at the University of Tehran, Iran. Basic culture media used in this study included YPGA and YDC, which were prepared using the procedure described in the literature (1).

### Semi-selective medium for *Clavibacter*

To develop a semi-selective medium, nine antibiotics, including amikacin, ciprofloxacin, colistin, imipenem, meropenem, nalidixic acid, neomycin, sulfamethoxazole, and trimethoprim, were screened for their inhibitory effect on bacterial growth. These antibacterial agents were selected based on their biochemical properties attesting to their selective activity against gram-negative bacteria (33). Each antibiotic was individually added to the YPGA medium at concentrations of 5.0, 10.0, 25.0, 50.0, 75.0, 100.0, and 150.0 mg/L to evaluate its ability to inhibit nontarget bacterial growth. Preliminary assays were conducted using a set of both gram-negative and gram-positive bacterial strains from *Bacillus*, *Chryseobacterium*, *Clavibacter, Curtobacterium*, *Dickeya*, *Enterobacter*, *Pantoea*, *Pseudomonas*, *Streptomyces*, and *Xanthomonas* species (Table S1). The bacterial strains were individually streaked onto YPGA medium, including each of the above-mentioned antibiotics. Inoculated plates were incubated at 25°C–27°C for 96 h and frequently monitored for bacterial growth based on the presence or absence of visible colonies. The antibacterial agents that inhibited the growth of all gram-negative bacteria and non-*Clavibacter* gram-positive bacteria were selected for the subsequent experiments. Two broad-spectrum fungicides*,* such as pentachloronitrobenzene (PCNB) and natamycin, were also evaluated as potential fungal inhibitors.

When concentrations of the selected antibiotics and fungicide agents were optimized individually, they were applied together in the basal YPGA and YDC media to assess their collective behavior on both gram-negative and gram-positive bacteria (Table S3). The new selective media resulting from the integration of the antibiotic agents into the basal YPGA and YDC media were named ClaYPGA and ClaYDC, respectively (Table S2). To assess if modification of the YPGA medium into ClaYPGA has any adverse effect on the growth speed, colony size, and morphology of *Clavibacter* species, freshly prepared cell suspensions of the type strains of *C. michiganensis* ICMP 2550^T^ and *C. nebraskensis* CFBP 2405^T^ were serially diluted in sterile distilled water (SDW) with a 1:1 ratio of the cell suspension and SDW. Then, 20 µL of the resulting cell suspensions were spread onto the ClaYPGA and ClaYDC media poured in 6 cm in diameter Petri dishes, and the plates were incubated at 25°C–27°C for 10 days. The number and morphology of colonies, such as size, shape, and color, were recorded in both basal and modified semi-selective media to gain a comparative insight.

### Specific PCR primers for *Clavibacter*

To find a strictly specific DNA fragment for members of *Clavibacter*, all available whole-genome resources of the genus deposited in the NCBI GenBank database were retrieved (*n* = 489 as of 14 October 2025) using the procedure recommended by the service provider. All available whole-genome resources of type strains of the *Microbacteriaceae* family—excluding *Clavibacter* members—were also retrieved from the NCBI GenBank database to be used in the subsequent analyses. All *Clavibacter* genomes were subjected to gene prediction using PRODIGAL v2.6.3. The predicted genomic areas obtained for each strain were compared against those of all other *Clavibacter* strains using BLASTN v2.16.0+ to identify common genomic regions across the genus. The genomic areas identified as common among *Clavibacter* strains were then used as queries in BLASTN v2.16.0+ searches against the local *Microbacteriaceae* database to identify DNA fragments that were conserved in *Clavibacter* but absent in other members of the *Microbacteriaceae* family. This approach led to the identification of *Clavibacter*-specific genomic regions suitable for specific primer design. A 259-bp DNA fragment was selected and subjected to further analyses. BLASTn on the NCBI nucleotide and whole-genome shotgun sequence databases confirmed the specificity of this fragment to *Clavibacter* members (Fig. S3). Thus, nucleotide sequences of the latter 259-bp DNA fragment were retrieved from all 489 *Clavibacter* strains, aligned using the MAFFT v.7 alignment tool (34), and visualized with MEGA software (35). A pair of PCR primers covering all 259-bp length of the target DNA was designed manually, while physical parameters of the primers were calculated using the NCBI Primer BLAST service.

To assess the specificity of the primer pair to *Clavibacter* members, they were subjected to conventional PCRs using a collection of plant-associated bacteria covering both gram-negative and gram-positive members (Table S3). For all bacterial strains, DNA was extracted from a freshly grown culture on YPGA medium for 48 h at 27°C, using the boiling method. For each strain, a single colony of bacterial culture was suspended in SDW and heated in a microfuge tube for 5 min in the boiling water bath. The prepared DNA suspensions were kept at −20°C for further use. For PCR reactions, Universal PCR Kit, Ampliqon Taq DNA Polymerase Master Mix Red (Ampliqon A/S, Odense, Denmark) was applied according to the manufacturer’s recommendations. For each strain, a 25-µL PCR, including 2 µL of total DNA and 2 µL of each primer (10 pmol/µL), was used. The annealing temperature was set to 65°C. All PCR reactions were repeated twice.

### Combination of semi-selective medium and specific PCR

Both semi-selective media (ClaYPGA and ClaYDC) and specific PCR primers produced satisfactory results for the isolation and identification of *Clavibacter* members, respectively. To evaluate the collective efficiency of these two techniques, two independent experiments were conducted as follows.

First, naturally infected tomato and alfalfa plants colonized with *C. michiganensis* and *C. insidiosus*, respectively, were used for the isolation of the corresponding bacterial pathogens on ClaYPGA and ClaYDC media using the procedure described previously (27). In brief, symptomatic pieces of each sample were cut using a sterile scalpel and macerated in 10*–*20 mL of SDW. A loopful of the resulting suspension was streaked onto YPGA, YDC, ClaYPGA, and ClaYDC media. The plates were incubated at 25°C*–*27°C for 5–7 days post-incubation, and bacterial colonies representing the entire range of colony morphology were purified by repetitive streaking on fresh YPGA plates. Purified strains were re-suspended in SDW and subjected to DNA extraction and PCR using the newly designed primer pairs. The PCR procedure was the same as described above.

In the second experiment, plant and soil specimens collected from fields of vegetables, annual crops, and ornamentals were subjected to bacterial isolation on ClaYPGA and ClaYDC media, as well as the basal YPGA and YDC media. The specimens included samples from aerial parts, roots, and rhizosphere soil of the corresponding plants, as detailed in Fig. S4. The plant specimens were treated in the same manner as described above, while the soil samples were diluted in SDW to have a reasonable concentration of suspensions as recommended by Schaad et al. (14). The resulting suspensions were streaked onto YPGA, YDC, ClaYPGA, and ClaYDC media, and the plates were incubated at 25°C*–*27°C for 5*–*7 days post-incubation. Single bacterial colonies suspected to be members of coryneform bacteria were sub-cultured on YPGA medium and subjected to a PCR test as described above. Screening the corynebacterial colonies in the latter assays resulted in the identification of an orange-pigmented *Clavibacter* strain isolated from cucumber leaves. To further confirm the identity and taxonomic position of this strain, it was subjected to phylogenetic analyses using the procedure described previously (36). In brief, the *gyrB* gene of the latter strain was sequenced using the 2F/6R primer pair (37), and the resulting 977 bp DNA fragment was phylogenetically compared with those of all *Microbacteriaceae* members to confirm its precise taxonomic position.

## SUPPLEMENTAL MATERIAL

Supplemental material aem.00871-26-s0001.pdf Fig. S1 to S5; Tables S1 to S3.

ASM does not own the copyrights to Supplemental Material that may be linked to, or accessed through, an article. The authors have granted ASM a non-exclusive, world-wide license to publish the Supplemental Material files. Please contact the corresponding author directly for reuse.
