# Supplementary material for: Isolation and identification of Clavibacter made easy
Source: Appl Environ Microbiol. 2026 Sep 22;92(10):e00871-26. doi: 10.1128/aem.00871-26 (PMC13689487; doi:10.1128/aem.00871-26)
Supplement: Supplementary file 1 [file aem.00871-26-s0001.pdf]

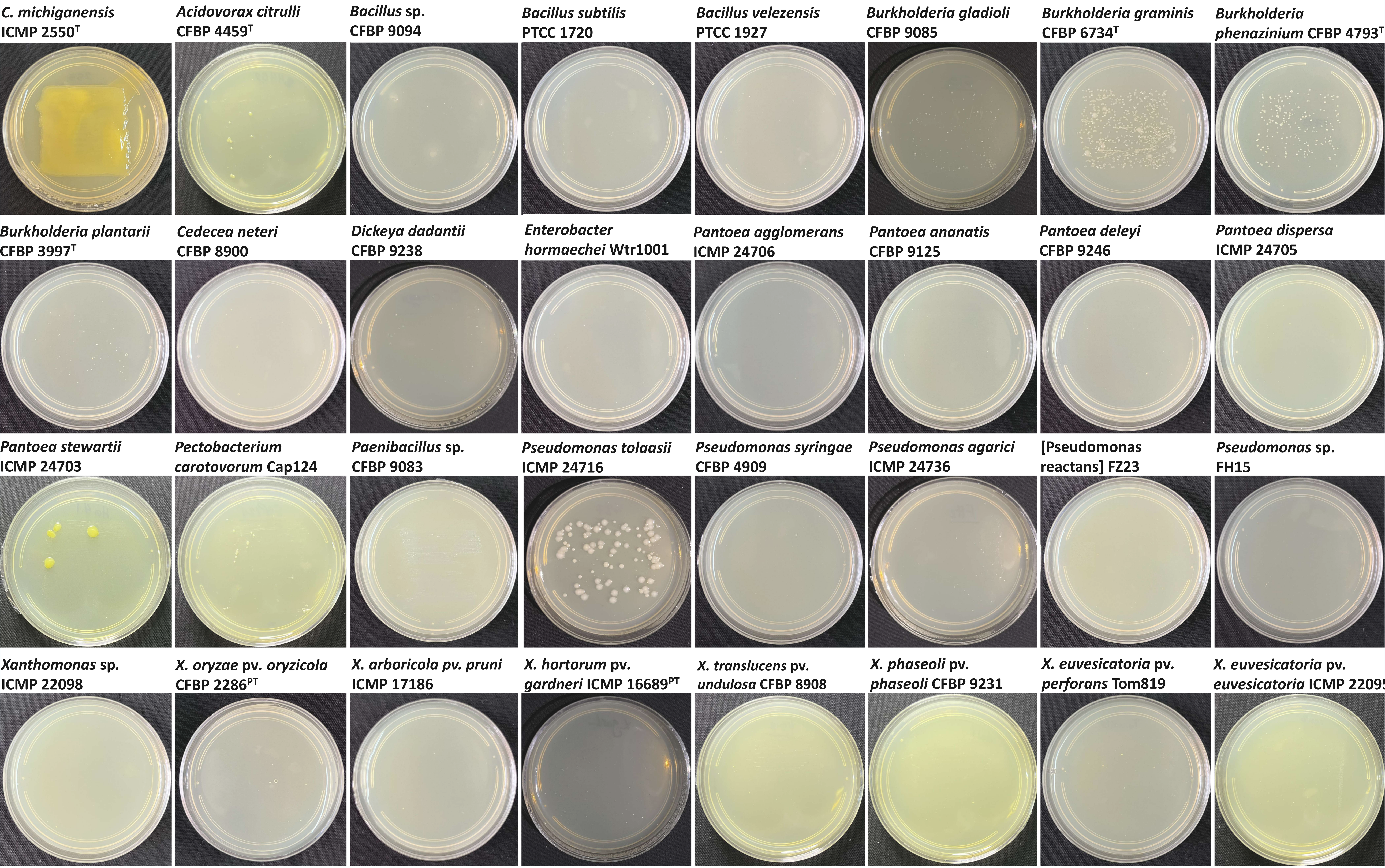

Figure S1: Growth pattern of Gram-negative plant associated bacteria on ClaYPGA medium incubated at 25-27 °C for 5-7 days. Clavibacter michiganensis ICMP 2550T is included as control.

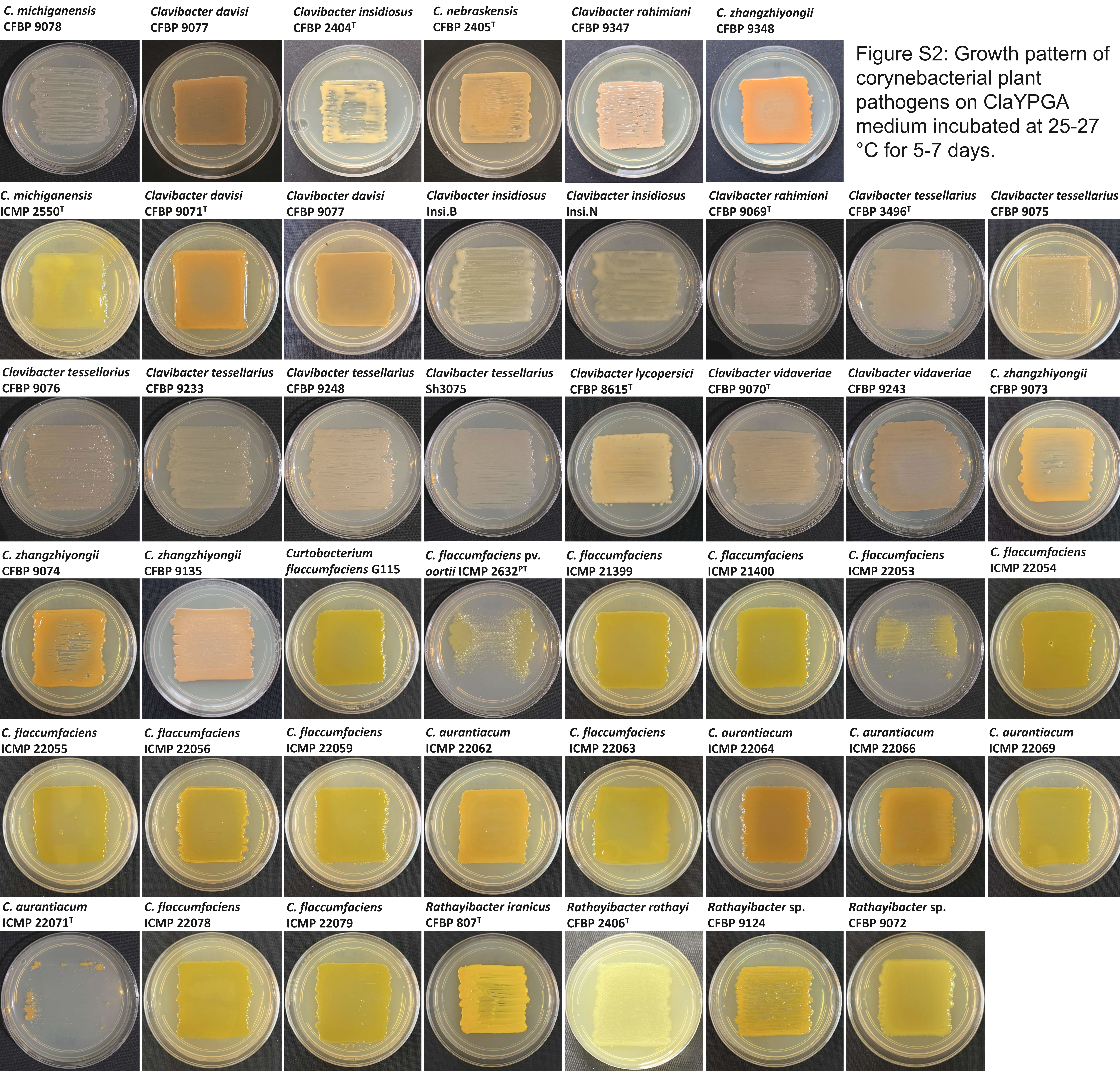

Figure S2: Growth pattern of corynebacterial plant pathogens on ClaYPGA medium incubated at 25-27 °C for 5-7 days.

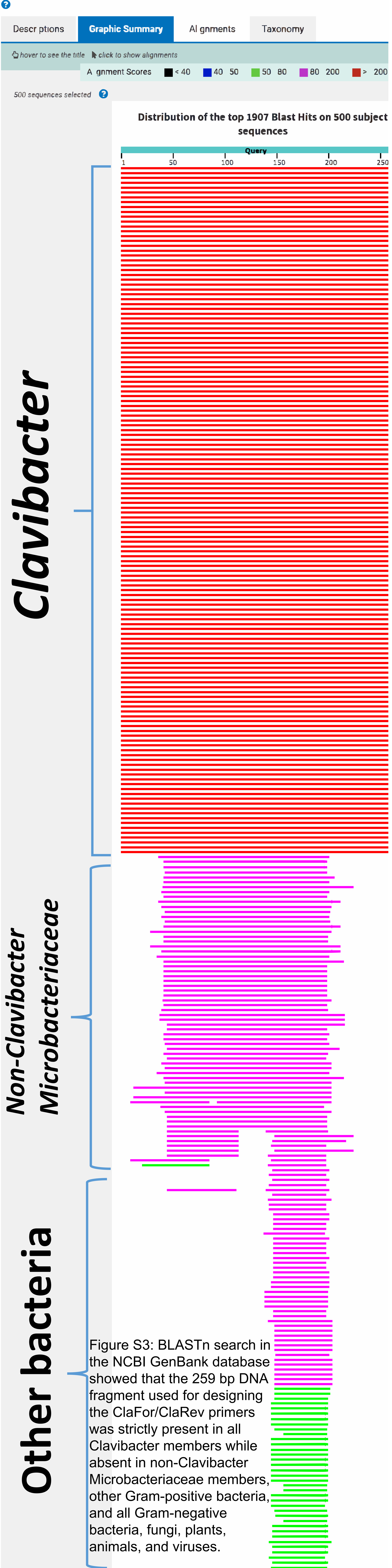

Figure S3: BLASTn search in the NCBI GenBank database showed that the 259 bp DNA fragment used for designing the ClaFor/ClaRev primers was strictly present in all Clavibacter members while absent in non-Clavibacter Microbacteriaceae members, other Gram-positive bacteria, and all Gram-negative bacteria, fungi, plants, animals, and viruses.

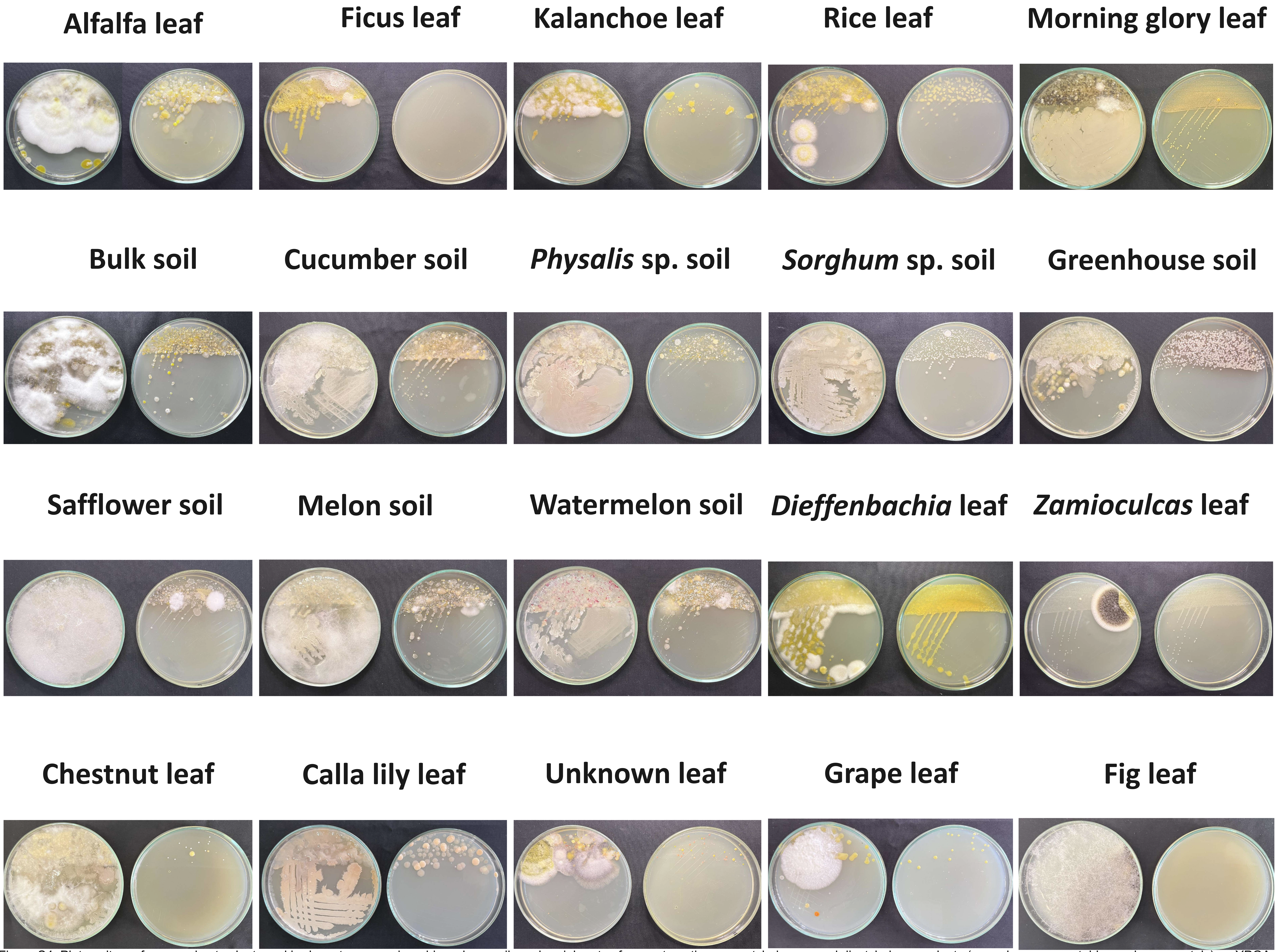

Figure S4: Plate cultures from non-hosts plants and irrelevant sources, i.e., rhizosphere soils and aerial parts of asymptomatic monocotyledonous and dicotyledonous plants (annual crops, vegetables and ornamentals) on YPGA (left side plates) and ClaYPGA (right side plates) for isolation of putative Clavibacter strains. An orange pigmented Clavibacter strain was isolated from cucumber leaves as illustrated in Figure 3C.

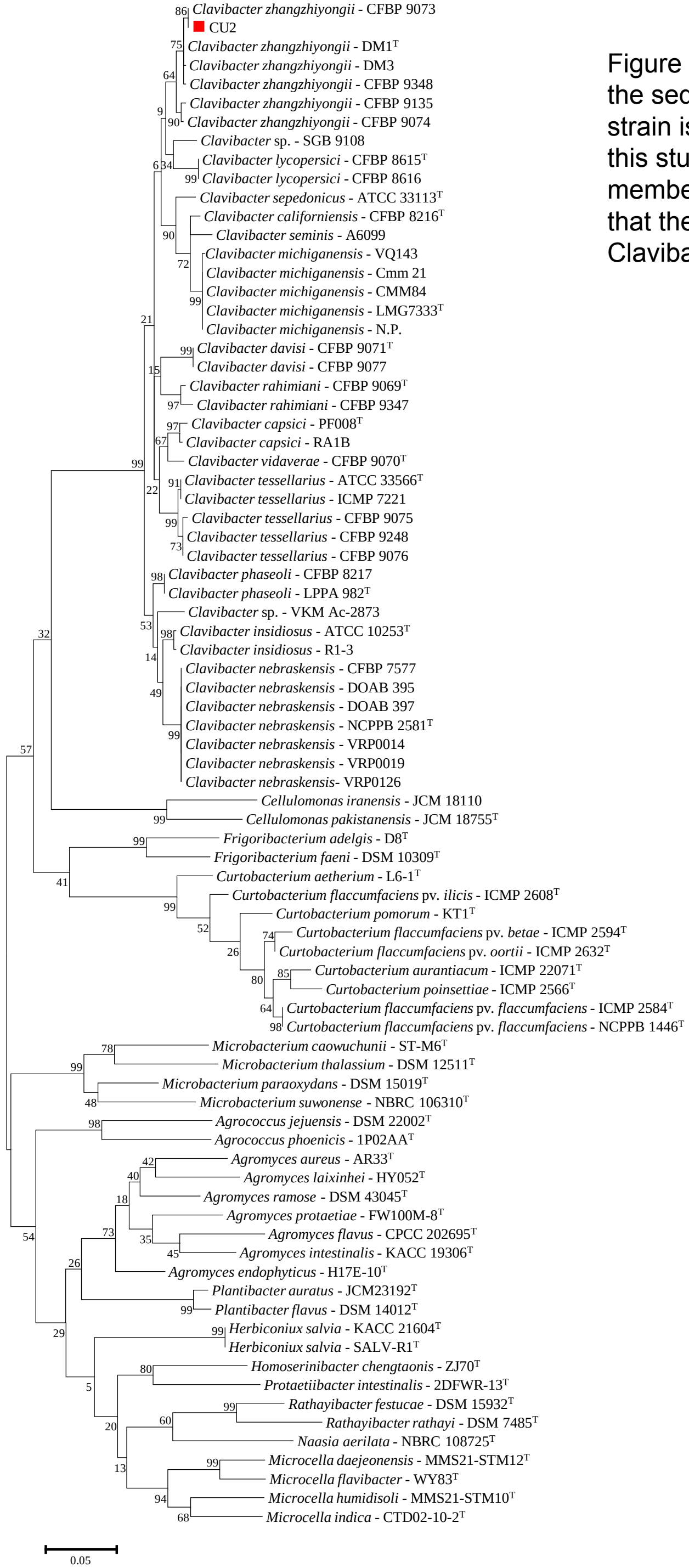

Figure S5: Phylogenetic analyses using the sequences of gyrB gene in the strain isolated from cucumber leaves in this study (CU2) and representative members of Microbacteriaceae showed that the strain CU2 belonged to Clavibacter zhangzhiyongii.

Table S1: Antibiotics used in this study and the results of bacterial growth test using different concentrations.

Bacterial species	Gram reaction	Antibiotics								
		Colistin			Nalidixic acid			Sulfamethoxazole / Trimethoprim		
		25 mg/lit	50 mg/lit	Trimethoprim	10 mg/lit	25 mg/lit	50 mg/lit	10 mg/lit	25 mg/lit	50 mg/lit
C. michiganensis	G^+	+	+	+	+	+	±	+	–	±
C. tessellarius	G^+	+	+	+	+	+	+	+	+	+
C. zhangzhiyongii	G^+	+	+	+	+	±	±	+	+	+
C. insidiosus	G^+	+	+	+	+	±	+	+	+	+
Curtobacterium spp.	G^+	+	+	+	+	±	+	+	–	±
Streptomyces spp.	G^+	+	+	+	+	–	+	+	+	+
Bacillus spp.	G^+	+	+	+	+	±	–	±	–	–
Xanthomonas spp.	G^-	±	–	–	–	–	–	±	±	–
Pseudomonas spp.	G^-	±	±	–	±	–	+	+	±	–
Pantoea spp.	G^-	±	±	–	–	–	–	±	–	–
Dickeya spp.	G^-	+	+	+	–	–	–	±	–	–
Chryseobacterium spp.	G^-	+	+	+	–	–	–	+	+	+
Enterobacter spp.	G^-	±	±	–	±	–	+	±	±	±

+ = growth	± = slight growth	– = no growth

Table S2: Ingredients of the ClaYPGA/ClaYDC semi-selective media developed in this study.

ClaYPGA		ClaYDC	
**Basal reagents**			
**Ingredient**	**Concentration**	**Ingredient**	**Concentration**
Yeast extract	5.0 g/L	Yeast extract	10.0 g/L
Peptone	5.0 g/L	Dextrose/glucose	20.0 g/L
Glucose	10.0 g/L	Calcium carbonate	20.0 g/L
Agar	15.0 g/L	Agar	15.0 g/L
**Antimicrobial reagents**			
**Ingredient**	**Concentration**	**Ingredient**	**Concentration**
Colistin	100.0 mg/L	Colistin	100.0 mg/L
Nalidixic acid	5.0 mg/L	Nalidixic acid	5.0 mg/L
Sulfamethoxazole	5.0 mg/L	Sulfamethoxazole	5.0 mg/L
Trimethoprim	5.0 mg/L	Trimethoprim	5.0 mg/L
Natamycin	500.0 mg/L	Natamycin	500.0 mg/L
All ingredients are dissolved in 1,000 mL of distilled water.			

Table S3: Full list of the bacterial strains used in this study, their origine, and results of their growth on ClaYPGA/ClaYDC medium and PCR using ClaFor/ClaRev primers.

Taxon	Strain	Host of isolation	Pathogenic on	Colony color	Growth on ClaYPGA and ClaYDC	Clavibacter-specific PCR
**Clavibacter strains**						
Clavibacter michiganensis	ICMP 2550^T	Tomato	Tomato	Yellow	Positive	Positive
Clavibacter michiganensis	CFBP 9078	Tomato	Tomato	Pink	Positive	Positive
Clavibacter insidiosus	CFBP 2404^T	Alfalfa	Alfalfa	Yellow	Positive	Positive
Clavibacter nebraskensis	CFBP 2405^T	Maize	Maize	Orange	Positive	Positive
Clavibacter lycopersici	CFBP 8615^T	Tomato	Non-pathogenic	Peach color	Positive	Positive
Clavibacter tessellarius	CFBP 9248	Wheat	Small grain cereals	Orange	Positive	Positive
Clavibacter tessellarius	CFBP 9075	Wheat	Small grain cereals	Orange	Positive	Positive
Clavibacter tessellarius	CFBP 9076	Wheat	Small grain cereals	Orange	Positive	Positive
Clavibacter tessellarius	CFBP 9233	Wheat	Small grain cereals	Orange	Positive	Positive
Clavibacter tessellarius	CFBP 9398	Wheat	Small grain cereals	Orange	Positive	Positive
Clavibacter tessellarius	Sh3075	Wheat	Small grain cereals	Orange	Positive	Positive
Clavibacter tessellarius	CFBP 3496^T	Wheat	Small grain cereals	Orange	Positive	Positive
Clavibacter zhangzhiyongii	CFBP 9073	Barley	Small grain cereals	Orange	Positive	Positive
Clavibacter zhangzhiyongii	CFBP 9074	Oat	Small grain cereals	Orange	Positive	Positive
Clavibacter zhangzhiyongii	CFBP 9135	Barley	Small grain cereals	Orange	Positive	Positive
Clavibacter zhangzhiyongii	CFBP 9348	Wheat	Small grain cereals	Orange	Positive	Positive
Clavibacter rahimiani	CFBP 9069^T	Wheat	Small grain cereals	Peach color	Positive	Positive
Clavibacter rahimiani	CFBP 9347	Wheat	Small grain cereals	Peach color	Positive	Positive
Clavibacter vidaverae	CFBP 9070^T	Wheat	Small grain cereals	Pale orange	Positive	Positive
Clavibacter vidaverae	CFBP 9243	Wheat	Small grain cereals	Pale orange	Positive	Positive
Clavibacter davisi	CFBP 9071^T	Oat	Small grain cereals	Deep yellow	Positive	Positive
Clavibacter davisi	CFBP 9077	Oat	Small grain cereals	Deep yellow	Positive	Positive
**Non-*Clavibacter* Gram-positive bacteria**						
Mycetocola saprophilus	CFBP 8708	Button mushroom	Button mushroom	Cream	Positive	Negative
Rathayibacter rathayi	CFBP 2406^T	Wheat	Small grain cereals	Yellow	Positive	Negative
Rathayibacter sp.	CFBP 9124	Maize	Not determined	Peach color	Positive	Negative
Rathayibacter iranicus	CFBP 807^T	Wheat	Small grain cereals	Yellow	Positive	Negative
Rathayibacter sp.	CFBP 9072	Wheat	Not determined	Yellowish orange	Positive	Negative
Rathayibacter sp.	CFBP 9136	Barley	Not determined	Yellowish orange	Positive	Negative
Rathayibacter tritici	CFBP 1385	Wheat	Not determined	Yellowish orange	Positive	Negative
Paenibacillus sp.	CFBP 9083	Saffron	Bulbous plants	Cream	Negative	Negative
Bacillus velezensis	PTCC 1927	Not determined	Not determined	Cream	Negative	Negative
Bacillus cereus	PTCC 1015	Not determined	Not determined	Cream	Negative	Negative
Bacillus subtilis	PTCC 1720	Not determined	Not determined	Cream	Negative	Negative
Paenibacillus polymyxa	PTCC 1021	Not determined	Not determined	Cream	Negative	Negative
Bacillus sp.	CFBP 9094	Sugarcane	Fleshy plant tissues	Cream	Negative	Negative
Curtobacterium flaccumfaciens pv. *flaccumfaciens*	ICMP 21399	Common bean	Edible dry beans	Yellow	Positive	Negative

Curtobacterium flaccumfaciens	ICMP 21400	Chilli pepper	Non-pathogenic	Yellow	Positive	Negative
Curtobacterium flaccumfaciens pv. *flaccumfaciens*	ICMP 22069	Common bean	Edible dry beans	Orange	Positive	Negative
Curtobacterium flaccumfaciens pv. *flaccumfaciens*	ICMP 22078	Bell pepper	Edible dry beans	Yellow	Positive	Negative
Curtobacterium aurantiacum	ICMP 22062	Tomato	Edible dry beans	Red	Positive	Negative
Curtobacterium flaccumfaciens pv. *flaccumfaciens*	ICMP 22054	Eggplant	Edible dry beans	Yellow	Positive	Negative
Curtobacterium flaccumfaciens pv. *flaccumfaciens*	ICMP 22055	Eggplant	Edible dry beans	Yellow	Positive	Negative
Curtobacterium flaccumfaciens	ICMP 22056	Eggplant	Non-pathogenic	Yellow	Positive	Negative
Curtobacterium flaccumfaciens pv. *flaccumfaciens*	ICMP 22057	Tomato	Edible dry beans	Yellow	Positive	Negative
Curtobacterium flaccumfaciens pv. *flaccumfaciens*	ICMP 22063	Tomato	Edible dry beans	Yellow	Positive	Negative
Curtobacterium flaccumfaciens pv. *oortii*	ICMP 2632[PT]	Tulip	Tulip	Yellow	Weakly Positive	Negative
Curtobacterium flaccumfaciens	ICMP 22084	Tomato	Non-pathogenic	Yellow	Positive	Negative
Curtobacterium aurantiacum	ICMP 22071	Common bean	Not determined	Red	Negative	Negative
Curtobacterium flaccumfaciens pv. *flaccumfaciens*	ICMP 22053	Bell pepper	Edible dry beans	Yellow	Weakly Positive	Negative
Curtobacterium flaccumfaciens pv. *flaccumfaciens*	G115	Tomato	Edible dry beans	Yellow	Positive	Negative
Curtobacterium flaccumfaciens pv. *flaccumfaciens*	ICMP 22079	Eggplant	Edible dry beans	Yellow	Positive	Negative
Curtobacterium flaccumfaciens pv. *flaccumfaciens*	ICMP 22059	Tomato	Edible dry beans	Yellow	Positive	Negative
Curtobacterium aurantiacum	ICMP 22066	Common bean	Edible dry beans	Red	Positive	Negative
Curtobacterium aurantiacum	ICMP 22064	Tomato	Non-pathogenic	Red	Positive	Negative
**Gram-negative plant-associated bacteria**						
Burkholderia gladioli	CFBP 9085	Garlic	Fleshy plant tissues	Cream	Weakly Positive	Negative
Burkholderia cepacia	CFBP 2227[T]	Not determined	Fleshy plant tissues	Cream	Positive	Negative
Burkholderia plantarii	CFBP 3997[T]	Not determined	Not determined	Cream	Negative	Negative
Burkholderia phenazinium	CFBP 4793[T]	Not determined	Not determined	Cream	Weakly Positive	Negative
Burkholderia pyrrocinia	CFBP 4794[T]	Not determined	Not determined	Cream	Positive	Negative
Burkholderia vietnamiensis	CFBP 4796[T]	Not determined	Not determined	Cream	Negative	Negative
Paraburkholderia graminis	CFBP 6734[T]	Not determined	Not determined	Cream	Weakly Positive	Negative
Burkholderia cenocepacia	CFBP 8617	Banana	Fleshy plant tissues	Cream	Weakly Positive	Negative
Cedecea neteri	CFBP 8900	Button mushroom	Button mushroom	Cream	Negative	Negative
Acidovorax citrulli	CFBP 4459[T]	Watermelon	Watermelon	Cream	Negative	Negative
Serratia marcesens	CFBP 9107	Squash	Squash	Red	Weakly Positive	Negative
Ewingella americana	CFBP 8897	Button mushroom	Button mushroom	Cream	Negative	Negative
Dickeya dadantii	CFBP 9238	Philodendron	Fleshy plant tissues	Cream	Negative	Negative
Xanthomonas citri pv. *citri*	CFBP 9251	Mexican lime	Mexican lime	Pale yellow	Negative	Negative
Xanthomonas phaseoli pv. *phaseoli*	CFBP 9231	Common bean	Common bean	Pale yellow	Negative	Negative
Xanthomonas oryzae pv. *oryzicola*	CFBP 2286[PT]	Rice	Rice	Pale yellow	Negative	Negative

Xanthomonas translucens pv. *undulosa*	CFBP 8908	Wheat	Small grain cereals	Pale yellow	Negative	Negative
Xanthomonas hortorum pv. *gardneri*	ICMP 16689[PT]	Tomato	Tomato	Pale yellow	Negative	Negative
Xanthomonas euvesicatoria pv. *perforans*	Tom819	Tomato	Tomato	Pale yellow	Negative	Negative
Xanthomonas euvesicatoria pv. *euvesicatoria*	ICMP 22095	Pepper	Pepper and tomato	Pale yellow	Negative	Negative
Xanthomonas arboricola pv. pruni	ICMP 17186	Not determined	Not determined	Pale yellow	Negative	Negative
Xanthomonas sp.	ICMP 22098	Not determined	Not determined	Pale yellow	Negative	Negative
Xanthomonas hortorum pv. pelargonii	ICMP 24701	Pelargonium	Pelargonium	Pale yellow	Negative	Negative
Pseudomonas agarici	ICMP 24736	Button mushroom	Button mushroom	Cream	Negative	Negative
Pseudomonas sp.	FH15	Button mushroom	Button mushroom	Cream	Negative	Negative
[Pseudomonas reactans]	FZ23	Button mushroom	Non-pathogenic	Cream	Negative	Negative
Pseudomonas tolaasii	ICMP 24716	Button mushroom	Button mushroom	Cream	Weakly Positive	Negative
Pseudomonas tolaasii	CFBP 2068[T]	Button mushroom	Button mushroom	Cream	Negative	Negative
[Pseudomonas reactans]	FZ24	Button mushroom	Button mushroom	Cream	Negative	Negative
Pseudomonas syringae pv. *actinidiae*	CFBP 4909	Not determined	Not determined	Cream	Negative	Negative
Pantoea stewartii	ICMP 24703	Maize	Maize	Yellow	Negative	Negative
Pantoea deleyi	CFBP 9246	Maize	Maize	Yellow	Negative	Negative
Pantoea ananatis	CFBP 9125	Maize	Maize	Yellow	Negative	Negative
Pantoea dispersa	ICMP 24705	Maize	Maize	Yellow	Negative	Negative
Pantoea agglomerans	ICMP 24706	Maize	Maize	Yellow	Negative	Negative
Pectobacterium carotovorum	Cap124	Potato	Potato	Cream	Negative	Negative
Enterobacter hormaechei	Wtr1001	Watermelon	Watermelon	Cream	Negative	Negative
Chryseobacterium sp.	CFBP 8748	Mushroom	Mushroom	Yellow	Weakly Positive	Negative
